# Atomic force microscopy recognition of protein A on *Staphylococcus aureus* cell surfaces by labelling with IgG–Au conjugates

**DOI:** 10.3762/bjnano.4.84

**Published:** 2013-11-11

**Authors:** Elena B Tatlybaeva, Hike N Nikiyan, Alexey S Vasilchenko, Dmitri G Deryabin

**Affiliations:** 1 Department of Microbiology, Orenburg State University, Pobedy Ave, 13, 460018, Orenburg, Russia; 2 Department of Biochemical Physics, Orenburg State University, Pobedy Ave, 13, 460018, Orenburg, Russia; 3 Institute of micro- and nanotechnologies of Orenburg State University, Pobedy Ave, 13, 460018, Orenburg, Russia; 4 Institute of Cellular and Intracellular Symbiosis, RAS, Pionerskaya str., 11, 460000, Orenburg, Russia; 5 All-Russian Research Institute of Beef Cattle, RAA, 9 Yanvarja str, 29, 460000, Orenburg, Russia

**Keywords:** atomic force microscopy, IgG–gold nanoparticle conjugates, protein A, *Staphylococcus aureus*

## Abstract

The labelling of functional molecules on the surface of bacterial cells is one way to recognize the bacteria. In this work, we have developed a method for the selective labelling of protein A on the cell surfaces of *Staphylococcus aureus* by using nanosized immunogold conjugates as cell-surface markers for atomic force microscopy (AFM). The use of 30-nm size Au nanoparticles conjugated with immunoglobulin G (IgG) allowed the visualization, localization and distribution of protein A–IgG complexes on the surface of *S. aureus*. The selectivity of the labelling method was confirmed in mixtures of *S. aureus* with *Bacillus licheniformis* cells, which differed by size and shape and had no IgG receptors on the surface. A preferential binding of the IgG–Au conjugates to *S. aureus* was obtained. Thus, this novel approach allows the identification of protein A and other IgG receptor-bearing bacteria, which is useful for AFM indication of pathogenic microorganisms in poly-component associations.

## Introduction

The development of fast and sensitive methods for bacterial recognition remains an important problem in microbiology. In some cases the recognition includes the labelling of cells with different kinds of markers, which is followed by microscopy. In optical microscopy, immunochemical or immunofluorescent labels are used [1]. In the case of electron microscopy, specific antibodies are conjugated with electron-dense particles, such as colloidal gold [2]. Significant progress in microscopic techniques has been reached with the invention of the atomic force microscope (AFM) [3]. However, appropriate approaches for the utilization of AFM in revealing markers are still being developed.

Compared to traditional methods of visualization – scanning electron and optical microscopy – AFM offers important benefits: a high spatial resolution, a real quantitative data acquisition in three dimensions, a relatively simple and nondestructive sample preparation procedure and a flexibility in ambient operating conditions [4]. These benefits allow for the development of highly sensitive high-resolution methods for the detection of individual structures or labels on the surface of microorganisms. These, in turn, open wide prospects for the estimation of the exact quantity of bound markers, their topology on the surface and other kinds of immune and substrate-specific activity analyses. The prospective AFM approach uses a functionalized tip in order to obtain force curves for the protein-coated substrate and to measure the specific interaction forces [5–6]. The main restriction of this method is the requirement to use a liquid cell, which complicates the scanning process and often leads to the appearance of artefacts in the recorded images. AFM recognition of microorganisms can also include the detection of specific antigen/antibody (Ag/Ab) complexes on the surface of the cell wall. In this case, the detection process consists of a comparison of size distribution histograms of antigen molecules before and after their interaction with specific and nonspecific antibodies [7]. The application of this approach, however, is complicated by the existence of "noise" in the images caused by nonspecific interactions. These circumstances indicate the relevance of the development of a simple, sensitive and reproducible AFM recognition method that is available both for routine studies and for unambiguous interpretation of the AFM results. In order to increase the reliability of the complex detection on bacterial cell surfaces, specific proteins are conjugated with nanodimensional labels that are easily resolved by AFM and also have a distinct structure [7]. It, in turn, allows for the identification of interactions and the quantitative determination of the localization of resultant complexes. In order to evolve this technique of AFM recognition, our attention was also drawn to the possibility of identifying not only antigens, but also functional cell-surface receptors that bind host proteins and, therefore, are significant in the pathogenesis of infectious diseases. In particular, it is important to distinguish the cells that carry immunoglobulin-binding receptors on their surface: protein A produced by *Staphylococcus aureus* [8] and protein G expressed in group C and G *Streptococcus* bacteria [9]. These protein–protein interactions also lead to the formation of specific complexes on the cell surface, in which IgG molecules are bound in the wrong orientation (in relation to normal antibody function). Thus, bacteria are disrupted by opsonization [10] and phagocytosis [11].

In this regard, the aim of our work was the development of an AFM method to specifically label *Staphylococcus aureus*, which bears protein A, with IgG–Au conjugates by using the direct visualization of the labels on the bacterial cell surface as a criterion for identification.

## Results

**Figure 1:**
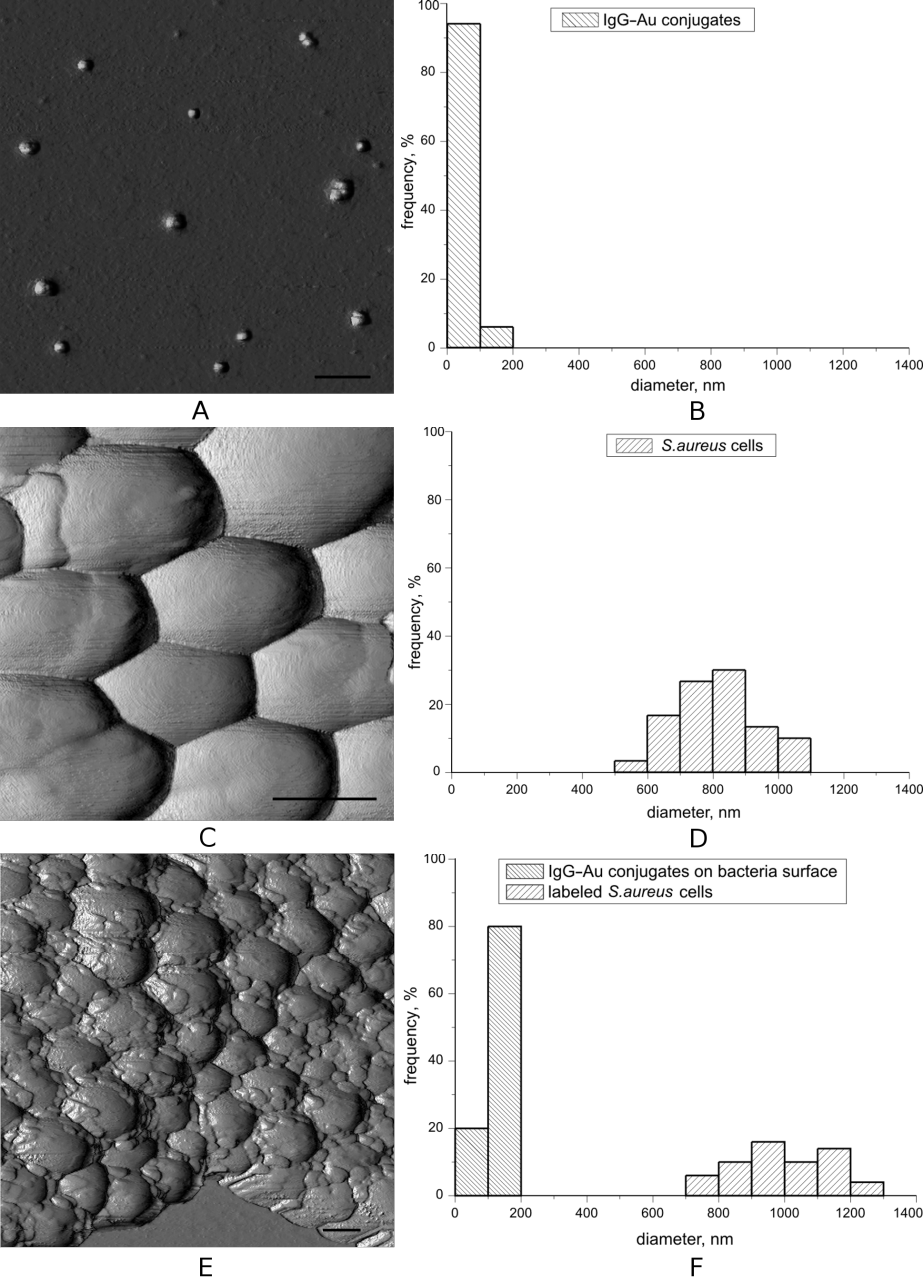
Topographic AFM images of the IgG–Au conjugates (a), *S. aureus* cells before (c) and after (e) contact with conjugates. Scale bar is 500 nm in all panels. (b), (d), (f) - Size (diameter) distribution histograms of corresponding structures. Information for each histogram was collected from several scans.

In the first step of our experimental procedure, IgG–Au conjugates were imaged. In Figure 1a, the results of these measurements are shown. Morphometric analysis showed that the average size (diameter) value of the observed structures was 80 ± 12 nm and had a small dispersion (Figure 1b). Considering the broadening effect caused by the tip, it can be concluded that conjugates are found on the mica surface both in single form and as aggregates composed of 2–3 conjugates. Taking into account the broadening effect of the tip, the observed size was significantly greater than the size of IgG that was estimated in [12]. This indicates that the conjugate size is mainly defined by gold nanoparticle dimensions. Under these experimental conditions, there was no aggregation of conjugates as indicated in [13]. These results were used as background for the following labelling and recognition of IgG–Au conjugates on the bacterial surfaces.

As visualized in the second step of the study, intact *S. aureus* cells appeared on the mica surface as grape-like clusters of round cocci. These formations occurred because of cells that remained attached to one another after dividing and were promoted by protein A, which induces bacterial aggregation in liquid media [14]. The diameter of cells observed in clusters (Figure 1c) varied from 600 to 1040 nm (Figure 1d); the average value was 800 ± 120 nm and was typical for this microorganism.

Analysis of the mean-square roughness (*R*_q_) of the *S. aureus* surface suggested that the bacteria have a relatively smooth surface (*R*_q_ = 1.03 ± 0.45 nm), typical for noncapsulated Staphylococcus cells [15]. The lack of a capsule is an important condition for the subsequent successful visualization of complexes of IgG–Au conjugates with protein A that is anchored to the peptidoglycan pentaglycine bridges in *Staphylococcus*.

The third step of the study included imaging *S. aureus* cells incubated with the IgG–Au conjugates. Formations with dimensions in the same range as the previously defined IgG–Au conjugates (100–253 nm) were identified on the cell surface (Figure 1e). However, the comparisons of the size distributions of the conjugates and the mentioned formations (Figure 1f) indicated a difference in the average values. The average diameter of aggregates observed on the bacteria was 140 ± 40 nm.

The size distribution histogram in Figure 1f shows that the formation of the complexes led to an increase in the cell diameters of *Staphylococcus*. The average diameter of the observed cells was 990 ± 140 nm and differed from intact cells with a high reliability (P < 0.0001). Roughness of the cell surface was higher values this time (*R*_q_ = 2.60 ± 2.23 nm).

**Figure 2:**
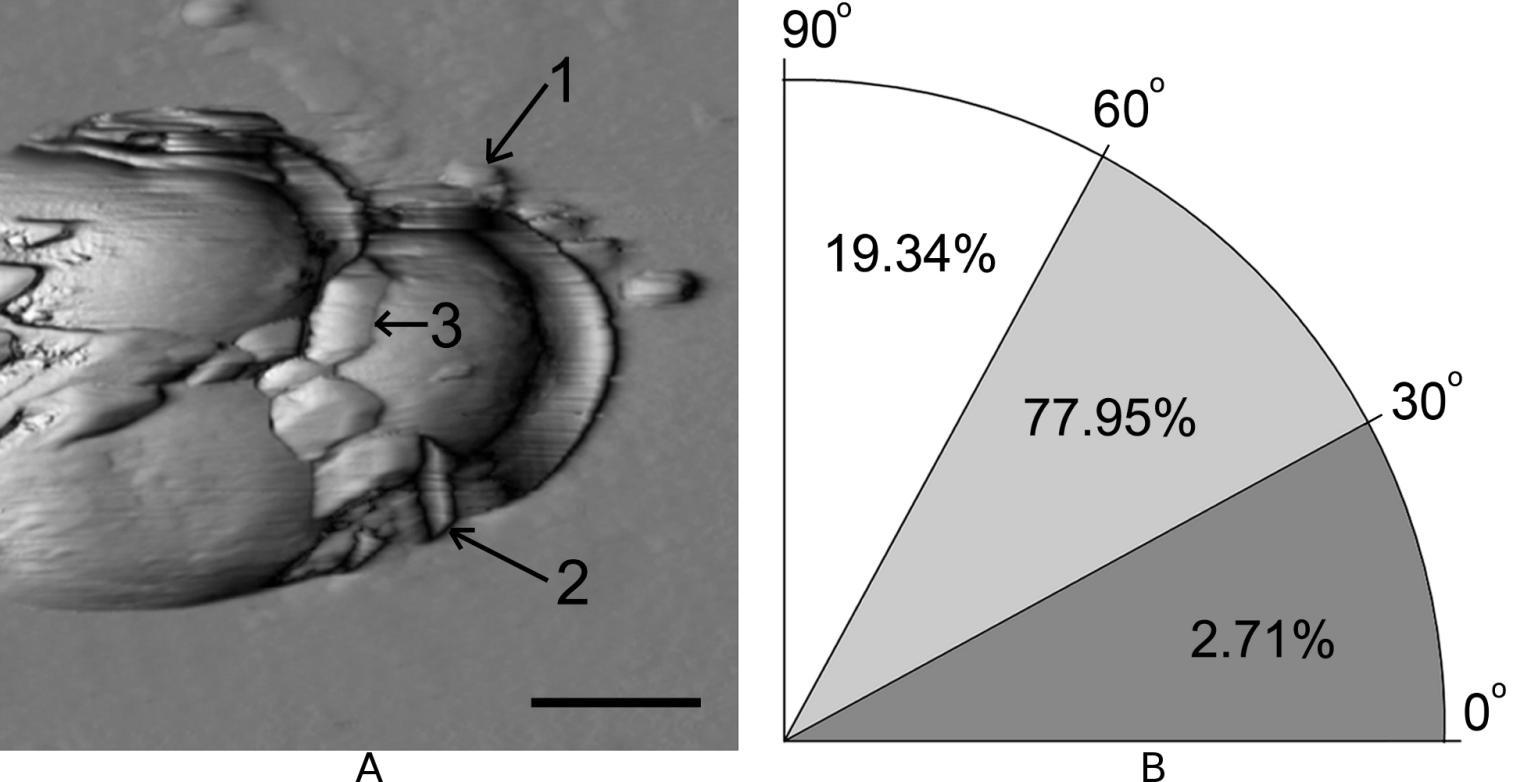
Distribution of IgG–Au conjugates on *S. aureus* cells: AFM image (a) and allocation of aggregates on a cellular surface according to binding area (b). Scale bar is 500 nm.

An irregular distribution of IgG–Au conjugates on the cellular surfaces was established. We defined three ways of orientation of the IgG–Au labels according to binding area (Figure 2a): 1 - on top of the cell (90–60º angle range), 2 - on one side (60–30º) and 3 - at the bottom (30–0º) as shown in Figure 2a. The analysis of over 200 labels showed that the majority of particles (78%) were located in the second zone, 19% of particles were in the first, and only 3% of the total number of particles were located in the third zone (Figure 2b).

After contact with IgG–Au conjugates structures on the surface of *S. aureus* were detected, which showed size characteristics that corresponded to to initial IgG–Au conjugates. We consider this as demonstration for the affinity of staphylococcal protein A (SpA) to bind in the Fc region of IgG. At the same time, the observed result was comparable to the immunolabelling methodology based on the affinity of SpA for IgG, which is applicable to either immunofluorescence observation using light microscopy or immunogold detection with electron microscopic techniques [16] on the one hand, and corresponds to conceptions of IgG preferentially binding to protein A-rich zones on the other [17].

**Figure 3:**
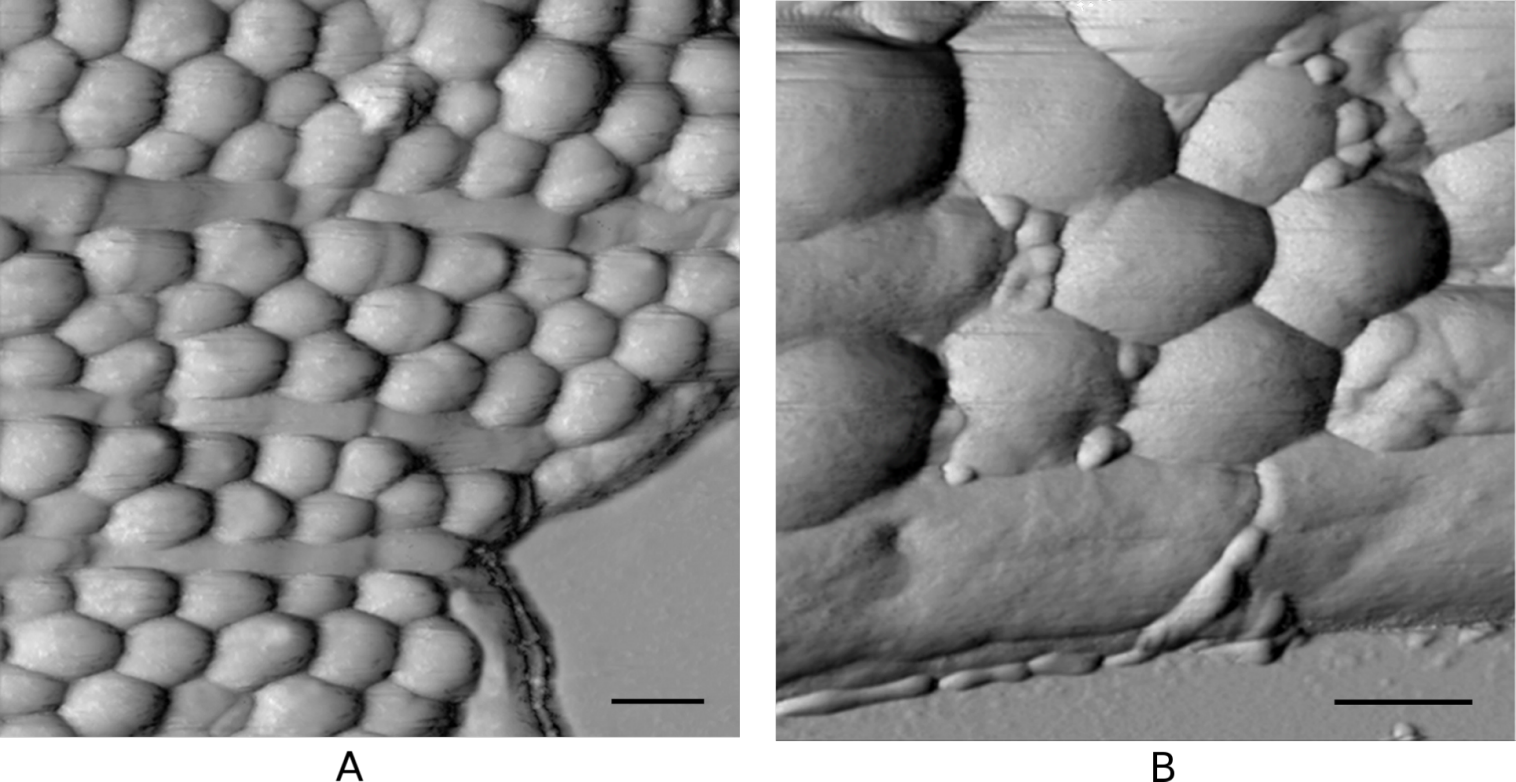
Topographic AFM images of *S. aureus* and *B. licheniformis* mixture before (a) and after (b) interaction with IgG–Au conjugates. Scale bar is 500 nm for both panels.

To confirm the selectivity of conjugates for *Staphylococcus* cells, mixes of bacteria that contained *S. aureus* and *B. licheniformis* incubated without and with IgG–Au conjugates were prepared. According to the shape, the type of cells can be easily distinguished in these mixes (Figure 3a). *Bacillus licheniformis* are rod-shaped bacteria 2.02 ± 0.12 µm in length and 0.91 ± 0.16 µm in width. In contrast to *S. aureus*, no protein A or other Fc receptors can be found on the surface of *B. licheniformis* [18], which suggests their inability of protein–protein interaction through the Fc region.

The result of co-incubation of *S. aureus* and *B. licheniformis* after the interaction with IgG–Au is shown in Figure 3b. After treatment with the conjugates, these bacterial cells were morphologically distinct and at the same time were differently labelled. On the surface of the *S. aureus* bacteria, IgG–Au conjugates were clearly visible (Figure 3b) and had the same size and arrangement as in the previous experimental series. However, the surface of *B. licheniformis* was clear or had a small quantity of particles bordering on the staphylococci area. The distribution of conjugates along these bacterial surfaces was then analysed. The majority of particles (66%) observed in the scan area were localized on the staphylococci surface, 5% were observed on the substrate, 19% were in the areas between the cells, and only 10% of the particles were observed on the surface of bacilli.

Thus, the preferable binding of IgG–Au conjugates to the surface of the protein A-positive *S. aureus* in contrast to the protein A-negative *B. licheniformis* was shown. However, absolute selectivity of binding was not established, which reduces the efficiency of differentiation and requires further research.

## Discussion

**Figure 4:**
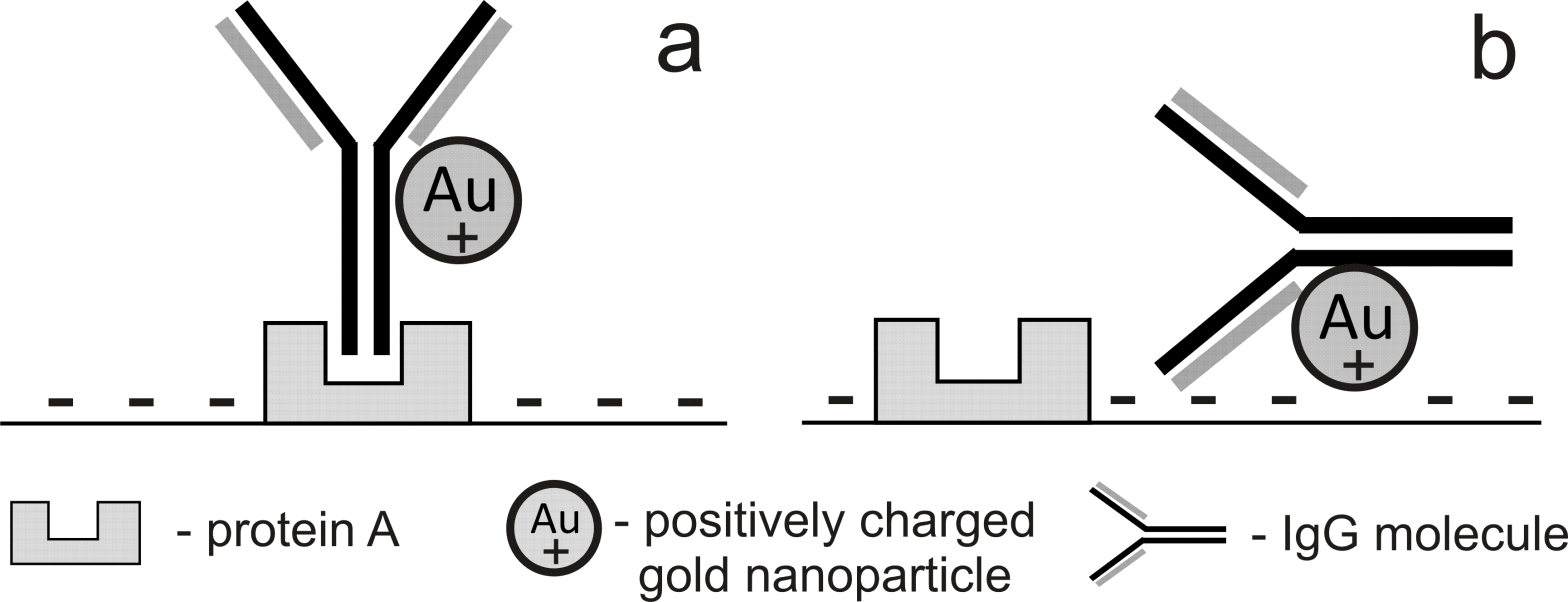
Two types of conjugate interactions with bacterial cell surfaces: selective binding of the Fc region of IgG in a non-antigenic way to protein A (a); non-selective electrostatic binding of colloidal gold to membrane (b).

The use of immunogold labels as cell-surface markers for atomic force microscopy was already offered during the early stages of the development of the method [19]. Meanwhile, even though the use of AFM is growing rapidly in microbiology and a number of different AFM techniques enable the study of biomaterials [4,20], gold labelling is still not a routine procedure. We are sure that the development of methods of nanogold synthesis with precise dimensional characteristics and shape [21], and also their conjugation with various functional proteins, is a key to the effective use of AFM for studying the interactions between single molecules, which includes protein–protein interactions and recognition, and also for solving specific cellular discrimination and AFM imaging problems. In this paper, we described the use of IgG–Au conjugates for marking *S. aureus* cells that bear protein A on the surface, which functions as an Fc receptor for immunoglobulins [22–23]. The essence of this suggested approach was the application of AFM to detect protein A–IgG complexes on the bacterial cell surfaces by using colloidal gold nanoparticles as labels (Figure 4a).

A moderate heterogeneity of the dimensional characteristics of the IgG–Au conjugates was revealed by AFM image analysis. Thus, conjugates were found both as single, spherically shaped objects, 80 ± 12 nm in size, and partially as aggregates composed of 2–3 particles. The registered size of the conjugates was defined by the size of nanogold particles and it was significantly larger than that of single, unlabeled IgG proteins that were estimated by X-ray diffraction analyses (14.2 nm) [13]. Because of their typical shape and size, these structures were easily distinguished by AFM and were very convenient for the use as labels.

The binding of individual IgG–Au conjugates to *S. aureus* bacterial cell walls led to the formation of aggregates on the cell surface. A further analysis showed an uneven distribution of aggregates over the staphylococcal surface, which can be explained by the deposition of protein A at discrete locations in the envelope [18]. It was a very promising result that confirmed the possible use of IgG–Au conjugates for protein A marking. Moreover, it provided an opportunity to detect bacterial cells that bear the protein, the estimation of the distribution of protein A on the surface, and, potentially, also for evaluating its quantity in a cellular wall.

To prove the selectivity of IgG–Au conjugates to only bind cells that bear to protein A, a mixture of *S. aureus* and *B. licheniformis* cells, which differed in shape and size was made. *B. licheniformis*, besides strong differences in morphology to *S. aureus*, have no protein A or other Fc receptors on the surface, which should allow for a clear distinction because of the IgG–Au conjugates only binding to *S. aureus* surfaces.

The obtained results indicated a preferential, but not exclusive (66% of the particles), binding of IgG to *S. aureus*, which was in accordance with the initial hypothesis. For 24% of the IgG–Au conjugates, the result was not clear as they were not bound to a cellular surface or were localized at the border between *S. aureus* and *B. licheniformis*. Some of the observed labels (10%) were found on bacilli surfaces, which was undesirable for the selective labelling and discrimination of protein A-positive and -negative cells. We suppose that this was a nonspecific binding that can be explained by electrostatic interactions between negatively charged cell surfaces [24] and positively charged particles of colloidal gold [25], as shown in Figure 4b. This result partially limits the use of nanogold particles in cell suspensions with a negative zeta-potential on the surface. Further research requires an improvement of label properties, in particular by neutralization of the surface charges of the gold nanoparticle by anionoid compounds.

## Conclusion

We herein present a method to recognise protein A-bearing *Staphylococcus aureus* by using IgG–Au conjugates as cell-surface markers and an AFM technique for their detection on bacterial surfaces. Because of the typical shape and size of colloidal gold nanoparticles, the localization of labels and their distribution on the bacterial surfaces can be studied. The prevalence of IgG–Au conjugates at cell division zones was demonstrated as well as their preferential binding to protein A-bearing *S. aureus* surfaces contrary to protein A-deficient *B. licheniformis* cells in the mixtures. Thus, in comparison with previously developed methods, this method, which is based on the direct observation of labelled cell surfaces, may be a new approach for the identification of microorganisms in complex bacterial mixtures.

## Experimental

### Bacteria preparation

Two bacterial strains were used: *Staphylococcus aureus* (FDA 209P, ATCC 6538) possessing a high level of protein A [26] and *Bacillus licheniformis* (ATCC 2336), which has a cell wall devoid of protein A. Both the microorganisms are Gram positive, which allows them to be processed and investigated in the same conditions; however, they differ in form and size, sufficient for their morphological differentiation.

Bacterial strains were cultured on LB agar (Sigma-Aldrich, USA) at 37 °C for 24 h and then washed with distilled water. Cells were harvested by centrifugation (1700*g*, 7 min) of the bacterial suspension and were subsequently diluted with distilled water to produce about 10^9^ viable cells per mL. Bacterial concentration was determined by measuring the A_640_ of the culture.

### Immunolabelling and AFM sample preparation

Mouse monoclonal immunoglobulin G (IgG) antibodies against a genus-specific antigen of Chlamydia species conjugated with gold nanoparticles (IgG–Au conjugates, VedaLab, France) were used for bacterial cell labelling. These antibodies did not cross react with *S. aureus* or *B. licheniformis*, therefore, their binding was specific for protein A. The IgG–Au conjugates were mixed with a suspension of *Staphylococcus aureus 209 P* or a mixture of *Bacillus licheniformis* cells at a ratio of 1:2 and incubated for 1 h at 37 °С with constant stirring on a thermostatic orbital shaker ST-3 (“Elmi”, Latvia). The labelled suspensions were then centrifuged for 5 min at 1700*g*, and the supernatant was discarded. The unbound IgG–Au conjugates were additionally washed twice through consecutive resuspensions with distilled water. Along with the test samples, IgG–Au conjugates, intact cells of *Staphylococcus aureus 209 P* and the mixture of *Bacillus licheniformis* were incubated and processed in the same conditions and used as a control samples. For the imaging of dried samples, a 2.5-μL droplet of bacterial suspension was applied to a freshly cleaved mica surface and left to dry in a humidity-controlled environment at 93% according to [27]. The mica surface is most commonly used for protein AFM imaging because of its hydrophilic character, its atomically flatness and the high affinity for proteins [28].

### Atomic force microscopy imaging

Images were collected by using an SMM-2000 atomic force microscope (JSC "Proton-MIET Plant", Russia) operated in contact mode. V-shaped silicon nitride cantilevers MSCT-AUNM from Veeco Instruments Inc. with a spring constant of 0.01 N/m were used. The typical radius of the MSCT-AUNM tip is approx. 10 nm, which is comparable to the size of the gold conjugates utilized in immunolabelling experiments.

## References

[1] Ramos-Vara J A (2005). Vet Pathol.

[2] Kuo J (2007). Electron Microscopy (Methods and Protocols).

[3] Binning G, Quate C F, Gerber C (1986). Phys Rev Lett.

[4] Dufrêne Y F, Hinterdorfer P (2008). Eur J Physiol.

[5] Lv Z, Wang J, Chen G, Deng L (2010). Nanoscale Res Lett.

[6] Creasey R, Sharma S, Gibson C T, Craig J E, Ebner A, Becker T, Hinterdorfer P, Voelckera N H (2011). Ultramicroscopy.

[7] Maluchenko N V, Agapov I I, Tonevitsky A G, Moisenovich M M, Savvateev M N, Tonevitsky E A, Bykov V A, Kirpichnikov M P (2004). Biofizika.

[8] Graille M, Stura E A, Corper A L, Sutton B J, Taussig M J, Charbonnier J-B, Silverman G J (2000). Proc Natl Acad Sci U S A.

[9] Sjöbring U, Björck L, Kastern W (1991). J Biol Chem.

[10] Bukharin O V, Deryabin D G, Brudastov Yu A (1994). Bull Exp Biol Med.

[11] Foster T, Baron S (1996). Staphylococcus. Medical Microbiology.

[12] Yu Y G, Xu R X, Jiang X D, Ke Y Q (2005). Chin J Traumatol (Engl Ed).

[13] Chen Y, Cai J, Xu Q, Chen Z W (2004). Mol Immunol.

[14] Merino N, Toledo-Arana A, Vergara-Irigaray M, Valle J, Solano C, Calvo E, Lopez J A, Foster T J, Penadés J R, Lasa I (2009). J Bacteriol.

[15] Tollersrud T, Berge T, Andersen S R, Lund A (2001). APMIS.

[16] Forsgren A, Sjöquist J (1966). J Immunol.

[17] Spratt B G (1975). Proc Natl Acad Sci U S A.

[18] DeDent A C, McAdow M, Schneewind O (2007). J Bacteriol.

[19] Putman C A J, de Grooth B G, Hansma P K, van Hulst N F, Greve J (1993). Ultramicroscopy.

[20] Liu S, Wang Y (2010). Scanning.

[21] Barnard A S, Young N P, Kirkland A I, van Huis M A, Xu H (2009). ACS Nano.

[22] Frank M B, Frank M B (2001). Antibody Binding to Protein A and Protein G beads. Molecular Biology Protocols.

[23] Boyle M D P (1989). Bacterial Immunoglobulin-binding Proteins.

[24] Dickson J S, Koohmaraie M (1989). Appl Environ Microbiol.

[25] Leff D V, Brandt L, Heath J R (1996). Langmuir.

[26] Gross G N, Rehm S R, Toews G B, Hart D A, Pierce A K (1978). Infect Immun.

[27] Nikiyan H, Vasilchenko A, Deryabin D (2010). Int J Microbiol.

[28] Ouerghi O, Touhami A, Othmane A, Ouada H B, Martelet C, Fretigny C, Jaffrezic-Renault N (2002). Biomol Eng.

